# Diagnostic Technology: Trends of Use and Availability in a 10-Year Period (2011–2020) among Sixteen OECD Countries

**DOI:** 10.3390/healthcare11142078

**Published:** 2023-07-20

**Authors:** Manuela Martella, Jacopo Lenzi, Maria Michela Gianino

**Affiliations:** 1 Department of Public Health Science and Pediatrics, University of Turin, 10126 Torino, Italy; mariola.gianino@unito.it; 2 Department of Biomedical and Neuromotor Sciences, University of Bologna, 40126 Bologna, Italy; jacopo.lenzi2@unibo.it

**Keywords:** diagnostic technologies, diagnostic imaging, healthcare resources, allocative efficiency, priority setting

## Abstract

Background. Overuse of imaging results in cost increases, with little to no benefit to patients. The purpose of this study is to evaluate imaging tests and radiology equipment over a ten-year period in 16 Organisation for Economic Co-operation and Development (OECD) countries. Methods. Twelve countries were included in a time-trend analysis based on OECD indicators on diagnostic imaging (computer tomography [CT], magnetic resonance imaging [MRI], and positron emission tomography [PET]). These annual indicators included the number of exams per 1000 population, the number of devices per million population, and the number of exams per device. Average annual percent change was used to measure country-specific trends. Results. Most countries saw a rise in the exam-to-scanner ratio for CT, MRI, and PET, demonstrating a faster increase in exam volume than device volume. Italy exhibited an increase in CT, MRI, and PET equipment units during the same period, but not in exams, most likely due to a reduction in medical procedures during the pandemic. Only in Luxemburg, CT and PET examinations increased despite a reduction in scanners. Conclusions. Considering the expected increasing demand for diagnostics due to the evolving needs of the population, proper governance and resource allocation are necessary requirements for cost-efficient health systems.

## 1. Introduction

Diagnostic technologies have become a policy issue, and the three common diagnostic imaging technologies in OECD countries—magnetic resonance imaging (MRI), computed tomography (CT), and positron emission tomography (PET)—are no exception. Diagnostic technologies strongly support physicians and enhance clinical practice from screening to prognosis for detecting diseases at an early stage and monitoring them with increasing accuracy. Besides the advantages of better health outcomes, prompt access to diagnostic tests also helps to limit healthcare costs due to late-stage, invasive, and even superfluous treatments. However, some effects of diagnostic technologies can be seen as negative. There is copious literature regarding the inappropriate and excessive use of diagnostic tests [1,2,3]. Overuse of tests, i.e., the delivery of tests with no clear benefit or when the potential harms outweigh the potential benefits, is not only a waste of finite healthcare expenditure by diverting resources from beneficial tests and treatments, but also subjects patients to low-value care [4]. The OECD reported that 10–34% of health service spending is potentially inappropriate, and is thus considered ineffective and a waste of healthcare resources. Worldwide, it is estimated that inappropriate or low-value imaging account for 20–50% of radiological examinations [5]. This issue has received great attention from the American Board of Internal Medicine (ABIM) Foundation such that, in the past decade, the organization promoted a new project named “Choosing Wisely” to quantify low-value care procedures and assess the effectiveness of the de-implementation interventions [6]. Another relevant effect of diagnostic technologies is that they are expensive, and their introduction into healthcare systems can drive up health spending [7,8]. No recommendations or benchmarks regarding the level standard of CT, PET, or MRI equipment units by population are currently validated across countries. Further, the number of devices is strictly related to the quality of assistance and the accessibility of services. In addition to cost increase for health systems, oversupply likely brings with it overuse or over-imaging, without real benefits for patients. Moreover, shortage of devices would lengthen waiting lists, resulting in a travel burden for users in terms of time and costs [9].

Certainly, when evaluating the effects of technologies, one cannot ignore that the standard of CT, PET, or MRI equipment units at the population level pertains to the degree of utilization of their production capacities. Indeed, diagnostic technologies can be a waste of healthcare resources if they are underutilized.

The current study aims to study the evolution in the annual number of diagnostic exams per equipment unit from 2011 to 2020 (ten years) in 16 OECD countries. In order to understand the reasons for country-specific trends in exam-to-scanner ratios, the annual number of exams per 1000 population and the annual number of scanners per million population are also analyzed.

## 2. Materials and Methods

This time-trend analysis was conducted using secondary data on diagnostic exams and medical technology from the online database OECD Health Statistics 2022 (https://stats.oecd.org/ (accessed on 17 November 2022)) during the ten-year period from 2011 to 2020. Data from before 2011 and after 2020 were discarded due to high proportions of missing values. In particular, the analysis was conducted on data referring to CT, MRI, and PET. The choice to focus on CT, MRI, and PET technologies was based on their widespread availability in healthcare facilities and their utility in diagnosing a broad range of pathologies, both for complicated and uncomplicated clinical conditions. Moreover, the OECD dataset provides data on healthcare utilization exclusively for CT, MRI, and PET exams.

CT scanner is a widely available imaging technique for fast and detailed view of internal organs and structures, through radiations emitted by an X-ray tube, which rotates around the patient and generates an X-ray beam. The acquired data are digitized by a computer to represent transverse sections of internal organs and structures, as bones and muscles. Compared to conventional radiography, CT has better definition, with superior contrast resolution and no superimposition of anatomic structures. In the OECD database, single-photon emission computed tomography is not counted among CT scanners [10].

As a non-invasive imaging technology, MRI can reproduce also three-dimensional anatomical images for diagnosis and evaluation of diseases. MRI systems produce a strong external magnetic field through nonionizing radiofrequency radiation for induction, excitation and subsequent relaxation of protons or hydrogen atoms. An important tool in the assessment of diseases, MRI techniques enhance tissue contrast and multiplanar imaging capability for equal or superior imaging in several body regions, complementary to CT techniques [11,12].

PET is a highly specialized imaging technique based on short-lived radioactive substances that produces 3D images that are mainly used for assessing the spread of cancer in a patient’s body (PET-CT systems that use image fusion are counted among PET scanners in the OECD database).

For each of the three medical technologies described above, the following OECD indicators were considered:

(a) Annual number of exams per 1000 population, including exams provided in all hospitals and ambulatory care providers;
(b) Annual number of scanners per million population, including equipment used in all hospitals and ambulatory care settings;
(c) Annual number of exams per scanner, i.e., the ratio between rates (a) and (b).

Indicator (c) is the primary outcome of the study, while indicators (a) and (b) are secondary outcomes that constitute indicator (c) and help its interpretation. Specific OECD definitions, sources, and methods for all indicators, overall and for each member country, can be accessed from the OECD Health Statistics 2022 online platform or directly on the following links: http://stats.oecd.org/wbos/fileview2.aspx?IDFile=8d3b4c1f-0ccd-4d36-954a-9a81bea235d0 (accessed on 17 November 2022); http://stats.oecd.org/wbos/fileview2.aspx?IDFile=610ccb97-615e-451f-859b-6ab1b6eb0f6f (accessed on 17 November 2022); http://stats.oecd.org/wbos/fileview2.aspx?IDFile=08b7f999-9e5d-427d-b072-df4d3a3036e0 (accessed on 17 November 2022); http://stats.oecd.org/wbos/fileview2.aspx?IDFile=7a032f96-afc1-4e16-9fe0-d58fa2bbe944 (accessed on 17 November 2022).

The OECD indicators cover all exams and equipment units in hospitals and ambulatory care settings. Data stratified by setting (hospitals versus ambulatory care providers) were not analyzed due to the large number of missing values and inconsistencies in data collection and reporting across countries.

Countries with more than three years of missing data for CT, MRI, or PET use over the study period were discarded, leaving 16 OECD members for analysis: 12 European countries (Belgium, Czechia, Finland, France, Greece, Italy, Lithuania, Luxembourg, the Netherlands, Poland, Slovakia, Spain) and 4 non-European countries (United States, Israel, South Korea, Australia). CT data for Belgium before 2013 corresponded to the number of hospitals with scanners rather than the actual number of scanners and, thus, were removed from the analysis. No imputation of missing country-years was performed. Hence, only complete yearly data from each country contributed to the analysis.

To depict the temporal trends of OECD indicators for each study country, we performed a second-order Poisson autoregressive analysis accounting for nonlinear trends by building restricted cubic splines with the knot locations recommended by Harrell [13,14]. The number of knots was set to four because it provided the best visual trade-off between over- and under-smoothing of the time series.

Analysis of trends can be computed through joinpoint models, where several different lines are connected together at the inflection points; these models provide for quantification of time variations and identification of changes in trends. The average annual percent change (AAPC) represents the weighted average of the annual percent changes over a period of multiple years, and is used to summarize variations and, accordingly with joinpoint models, represent data as single numbers. Such models are useful to highlight trend transitions, for comparison of inconstant or variable trends over a time period. In the current study, the AAPC was used as a summary measure of country-specific trends in diagnostic exams and medical equipment. Heteroscedasticity and first-order autocorrelation of the random errors were considered for setting up the log-linear segmented regression. A maximum of one break point over the time series was settled on [15,16]. The 95% confidence intervals (CIs) of AAPCs were obtained adopting the empirical cumulative distribution function quantile method from Kim et al. [17].

In a sensitivity analysis, we replicated the calculation of AAPCs after removing 2020 from the time series to assess whether nonsignificant increases in diagnostic exams or medical equipment for some countries were attributable to the COVID-19 pandemic. Lastly, we checked the association between CT and MRI indicators over time using an entity-and-time fixed-effects linear regression model with clustered standard errors to deal with serial autocorrelation. Fixed effects were preferred over random effects because our analysis did not account for time-invariant variables, and time fixed effects were included in the model as dummies to control for the presence of exogenous time trends in both the dependent and independent variable (MRI and CT, respectively).

Data were managed, tabulated and displayed using Stata 17.0 (StataCorp. 2021. Stata Statistical Software: Release 17. College Station, TX, USA: StataCorp LLC), while AAPCs were obtained with Joinpoint Regression Program V.4.8.0.1 (April 2020; Statistical Methodology and Applications Branch, Surveillance Research Program, National Cancer Institute). Country-specific provisional values, deviations from international definitions, and changes in data source or methodology during the study period were reported as footnotes to charts and tables. The presence of such inconsistencies across country-years is the reason why no pooled estimates of exams per scanner were calculated by means of hierarchical regression modelling.

## 3. Results

### 3.1. Description

#### 3.1.1. Computed Tomography

**Figure 1:**
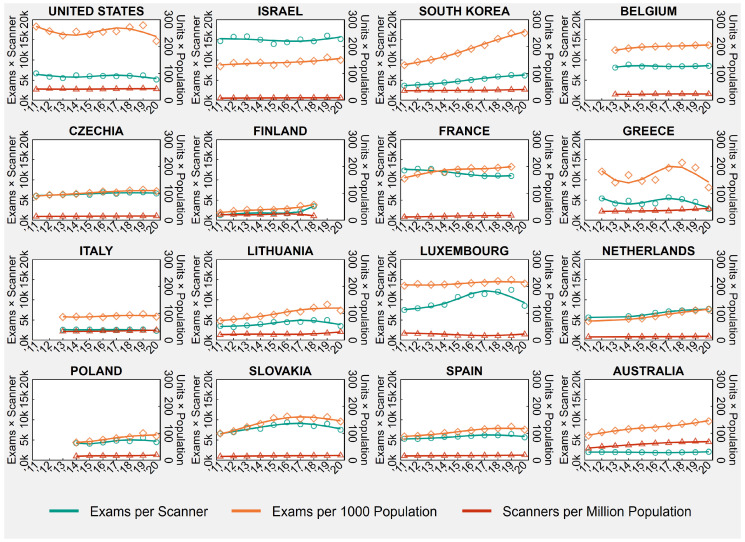
Computed Tomography (CT) Exams and Scanners in 16 OECD Countries, Years 2011–2020.

Notes: Overall rates observed in all country-years are displayed in the background of each plot, below population-averaged cubic splines. In France, before 2013, examinations practiced in private hospitals were wrongly compiled in ambulatory care, while during 2015, the official sources of data have been enhanced concerning all the equipment actually in use. In Poland, there was a change in data source in 2019 that led to the inclusion of specialty hospitals. In Australia, data relate to services rendered on a “fee-for-service” basis for which Medicare benefits were paid, which means that services that do not attract a Medicare benefit, such as those to public patients in hospitals or patients attending public Accident and Emergency Departments and public Outpatient Clinics, are excluded. Lastly, 2020 values for Luxembourg, the Netherlands, and Spain are provisional.

Abbreviations: OECD, Organisation for Economic Co-operation and Development.

Table 1 shows the country-specific number of CT exams per 1000 population and CT scanners per million population in the first and last available years between 2011 and 2020, as well the number of exams per scanner, obtained as ratio of the previously mentioned indicators. Table 1 also presents the country-specific AAPCs, while Figure 1 shows the trends of annual CT rates and ratios from 2011 to 2020.

**Table 1:** Computed Tomography (CT) Exams and Scanners in 16 OECD Countries, Years 2011 and 2020.

	2011 or First	2020 or Last	AAPC
	Available Year ^†^	Available Year ^‡^	(95% CI)
United States			
Exams per scanner	6695.4	5171.8	−0.7 (−1.8, 0.5)
Exams per 1000 population	273.8	220.2	−0.3 (−2.5, 2.0)
Scanners per million population	40.89	42.58	0.5 (−0.8, 1.8)
Israel			
Exams per scanner	14,668.7	15,183.2	0.1 (−1.1, 1.3)
Exams per 1000 population	126.6	149.9	1.7 * (0.3, 3.2)
Scanners per million population	8.63	9.88	1.5 * (1.2, 1.9)
South Korea			
Exams per scanner	3660.1	6159.4	6.7 * (6.0, 7.4)
Exams per 1000 population	131.0	250.0	7.9 * (7.3, 8.6)
Scanners per million population	35.79	40.59	1.2 * (0.8, 1.6)
Belgium			
Exams per scanner	8120.0	8541.2	0.1 (−0.6, 0.8)
Exams per 1000 population	186.3	205.0	1.4 * (1.3, 1.5)
Scanners per million population	22.94	24.01	1.1 * (0.4, 1.8)
Czechia			
Exams per scanner	6061.7	6643.4	1.3 * (0.8, 1.7)
Exams per 1000 population	89.5	108.1	2.5 * (1.8, 3.1)
Scanners per million population	14.77	16.26	1.2 * (0.9, 1.5)
Finland			
Exams per scanner	1308.8	3484.8	11.8 * (5.3, 20.4)
Exams per 1000 population	27.9	57.5	9.3 * (5.3, 14.2)
Scanners per million population	21.34	16.50	−0.1 (−3.8, 3.7)
France			
Exams per scanner	12,338.3	10,953.1	−2.1 * (−3.3, −0.9)
Exams per 1000 population	154.6	198.6	2.9 * (2.1, 3.7)
Scanners per million population	12.53	18.13	4.7 * (4.6, 4.8)
Greece			
Exams per scanner	5420.1	2786.9	−1.8 (−8.2, 5.2)
Exams per 1000 population	181.1	121.9	1.2 (−5.8, 9.2)
Scanners per million population	33.41	43.74	3.6 * (2.3, 4.9)
Italy			
Exams per scanner	2605.6	2333.3	−0.7 (−2.3, 0.9)
Exams per 1000 population	86.3	87.5	1.1 (−0.1, 2.4)
Scanners per million population	33.10	37.50	1.9 * (1.2, 2.6)
Lithuania			
Exams per scanner	3555.5	3557.8	2.0 (−0.2, 4.7)
Exams per 1000 population	71.6	110.7	6.4 * (4.9, 8.1)
Scanners per million population	20.14	31.13	3.9 * (2.1, 5.1)
Luxembourg			
Exams per scanner	7493.3	8521.4	2.9 * (1.7, 4.2)
Exams per 1000 population	202.1	210.4	0.8 * (0.3, 1.3)
Scanners per million population	25.08	22.21	−1.9 * (−3.4, −1.1)
Netherlands			
Exams per scanner	5654.5	7763.3	4.0 * (2.6, 5.8)
Exams per 1000 population	70.8	113.9	5.9 * (4.2, 8.4)
Scanners per million population	12.52	14.68	1.9 * (1.2, 2.6)
Poland			
Exams per scanner	4208.9	4479.4	4.2 * (2.7, 5.4)
Exams per 1000 population	65.8	89.9	7.1 * (6.0, 8.1)
Scanners per million population	15.63	20.08	3.0 * (2.0, 3.9)
Slovakia			
Exams per scanner	6602.3	7541.7	2.3 * (1.7, 2.9)
Exams per 1000 population	99.1	143.7	5.2 * (3.9, 6.9)
Scanners per million population	15.00	19.05	2.3 * (1.4, 3.2)
Spain			
Exams per scanner	5298.1	5657.0	1.5 * (1.0, 2.0)
Exams per 1000 population	88.2	113.3	3.4 * (3.0, 3.8)
Scanners per million population	16.64	20.04	1.8 * (1.7, 2.0)
Australia			
Exams per scanner	2057.7	2136.1	0.1 (−0.5, 0.6)
Exams per 1000 population	91.2	144.6	4.6 * (3.7, 5.5)
Scanners per million population	44.32	67.68	4.6 * (3.3, 6.4)

* The AAPC obtained from segmented regression analysis is significantly different from zero. ^†^ 2012 for Greece, 2013 for Italy, and 2014 for Poland. ^‡^ 2018 for Finland, and 2019 for France. Abbreviations: OECD, Organisation for Economic Co-operation and Development; AAPC, average annual per cent change; CI, confidence interval.

As shown in the time series in Figure 1, the number of CT exams and scanners varied greatly across OECD countries during the ten-year study period between 2011 and 2020. In 2020, or the last available year (Table 1), exams per 1000 population ranged from 57.5 in Finland to 250.0 in South Korea, scanners per million population ranged from 9.88 in Israel to 67.68 in Australia, and exams per scanner ranged from 2136.1 in Australia to 15,183.2 in Israel.

Between 2011 and 2020, the United States was the only country in the sample that did not experience any significant increase or decrease in CT exams and scanners, which resulted in a nonsignificant trend in exams per scanner (AAPC = −0.7, 95% CI = −1.8 to +0.5). Finland registered a significant increase only in CT exams (AAPC = +9.3, 95% CI = +5.3 to +14.2) that resulted in a significant increase in exams per scanner (AAPC = +11.8, 95% CI = +5.3 to +20.4), while Greece and Italy registered a significant increase only in CT scanners (Greece: AAPC = +3.6, 95% CI = +2.3 to +4.9; Italy: AAPC = +1.9, 95% CI = +1.2 to +2.6) that resulted in a nonsignificant trend in exams per scanner (Greece: AAPC = −1.8, 95% CI = −8.2 to +5.2; Italy: AAPC = −0.7, 95% CI = −2.3 to +0.9). Luxembourg experienced a significant increase in CT exams (AAPC = +0.8, 95% CI = +0.3 to +1.3) in conjunction with a significant decrease in the number of CT scanners (AAPC = −1.9, 95% CI = −3.4 to −1.1) (Table 1), which translated into a significant increase in exams per scanner (AAPC = +2.9, 95% CI = +1.7 to +4.2).

In the other 11 countries (Israel, South Korea, Belgium, Czechia, France, Lithuania, the Netherlands, Poland, Slovakia, Spain, Australia), both CT exams and scanners significantly grew in number between 2011 and 2020. The trend in the exam-to-scanner ratio was statistically nonsignificant in Israel, Belgium, Lithuania, and Australia, suggesting that in these countries, CT exams and scanners increased at a similar pace, while in South Korea, Czechia, the Netherlands, Poland, Slovakia, and Spain, the exam-to-scanner ratio increased significantly, suggesting that CT exams grew at a faster pace than scanners. Lastly, in France, the exam-to-scanner ratio decreased significantly (AAPC = −2.1, 95% CI = −3.3 to −0.9), suggesting that CT exams grew at a slower pace than scanners (Table 1).

Contrary to the main analysis, when 2020 was removed from the study period, we found that Italy experienced a significant increase in the number of CT exams before the outbreak of COVID-19 (AAPC = +1.8, 95% CI = +1.1 to +2.5) (Table S1 in the Supplementary Materials).

#### 3.1.2. Magnetic Resonance Imaging

**Figure 2:**
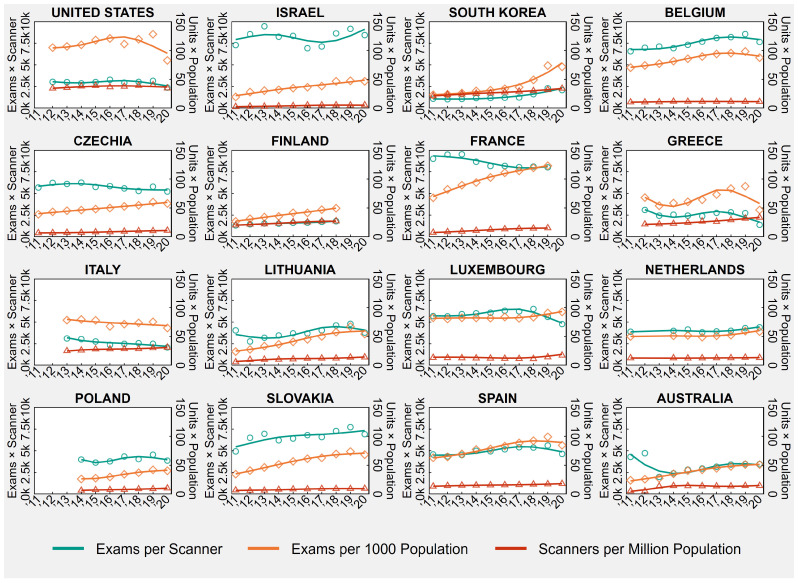
Magnetic Resonance Imaging (MRI) Exams and Equipment Units in 16 OECD Countries, Years 2011–2020.

Notes: Overall rates observed in all country-years are displayed in the background of each plot, below population-averaged cubic splines. In Israel, the increase in the number of MRI exams in 2018 is mainly due to an increasing number of hospitals reporting these data. In Belgium, since 2016 data are based on the national registry for devices of medical image and correspond to the number of MRI-devices. In France, before 2013, examinations practiced in private hospitals were wrongly compiled in ambulatory care, while during 2015, the official sources of data have been enhanced concerning all the equipment actually in use. In Poland, there was a change in data source in 2019 that led to the inclusion of specialty hospitals. In Australia, data relate to services rendered on a “fee-for-service” basis for which Medicare benefits were paid, which means that services that do not attract a Medicare benefit, such as those to public patients in hospitals or patients attending public Accident and Emergency Departments and public Outpatient Clinics, are excluded. Lastly, 2020 values for Luxembourg, the Netherlands, and Spain are provisional.

Abbreviations: OECD, Organisation for Economic Co-operation and Development.

Table 2 shows the country-specific number of MRI exams per 1000 population and MRI equipment units per million population in the first and last available years between 2011 and 2020, as well the number of exams per equipment unit, obtained as ratio of the previously mentioned indicators. Table 2 also presents the country-specific AAPCs, while Figure 2 shows the trends of annual MRI rates and ratios from 2011 to 2020. For reasons of consistency with the other tables and charts, MRI equipment units are named “scanners” in both Table 2 and Figure 2.

**Table 2:** Magnetic Resonance Imaging (MRI) Exams and Equipment Units in 16 OECD Countries, Years 2011 and 2020.

	2011 or First	2020 or Last	AAPC
	Available Year ^†^	Available Year ^‡^	(95% CI)
United States			
Exams per scanner	3042.1	2384.7	−0.5 (−2.3, 1.2)
Exams per 1000 population	104.8	82.7	0.5 (−2.1, 3.0)
Scanners per million population	34.46	34.66	0.8 (−1.3, 2.9)
Israel			
Exams per scanner	7276.2	8425.5	0.5 (−2.1, 3.8)
Exams per 1000 population	19.7	46.6	8.3 * (6.8, 10.2)
Scanners per million population	2.70	5.53	7.6 * (6.3, 8.7)
South Korea			
Exams per scanner	1095.6	2095.3	9.5 * (7.3, 11.9)
Exams per 1000 population	23.3	71.7	15.8 * (13.7, 17.8)
Scanners per million population	21.27	34.24	4.9 * (4.3, 5.6)
Belgium			
Exams per scanner	6564.6	7641.8	2.6 * (1.6, 3.6)
Exams per 1000 population	70.2	87.4	2.9 * (2.6, 3.1)
Scanners per million population	10.69	11.44	1.0 * (0.6, 1.4)
Czechia			
Exams per scanner	5678.8	5227.3	−1.5 * (−2.5, −0.6)
Exams per 1000 population	39.0	57.7	4.5 * (3.8, 5.2)
Scanners per million population	6.86	11.03	6.1 * (5.4, 6.8)
Finland			
Exams per scanner	1318.2	1809.6	4.0 * (3.0, 5.0)
Exams per 1000 population	26.7	49.5	8.5 * (7.6, 9.5)
Scanners per million population	20.23	27.38	4.6 * (3.9, 5.3)
France			
Exams per scanner	8991.9	8004.4	−2.2 * (−3.5, −0.6)
Exams per 1000 population	67.5	122.8	7.8 * (5.6, 10.0)
Scanners per million population	7.51	15.34	9.2 * (8.8, 9.5)
Greece			
Exams per scanner	3099.2	1384.7	−2.5 (−8.8, 4.8)
Exams per 1000 population	67.9	46.5	3.1 (−3.5, 10.7)
Scanners per million population	21.91	33.56	5.9 * (5.2, 6.7)
Italy			
Exams per scanner	3107.7	2071.1	−4.5 * (−7.4, −1.7)
Exams per 1000 population	78.3	64.7	−1.9 (−4.3, 0.5)
Scanners per million population	25.20	31.24	2.7 * (1.6, 3.9)
Lithuania			
Exams per scanner	4040.4	3787.4	4.4 * (2.0, 7.2)
Exams per 1000 population	24.0	54.2	11.1 * (9.0, 13.6)
Scanners per million population	5.94	14.31	6.1 * (3.6, 8.8)
Luxembourg			
Exams per scanner	5681.7	4768.9	−1.8 * (−1.9, −1.6)
Exams per 1000 population	82.5	92.5	1.6 * (1.1, 2.0)
Scanners per million population	13.50	17.45	3.5 * (3.3, 3.6)
Netherlands			
Exams per scanner	3880.9	4386.2	1.2 * (0.2, 2.1)
Exams per 1000 population	50.0	58.6	2.0 * (1.1, 2.7)
Scanners per million population	12.88	13.36	0.6 * (0.3, 1.0)
Poland			
Exams per scanner	3996.4	3872.5	2.3 * (0.6, 3.9)
Exams per 1000 population	26.4	40.6	9.1 * (7.4, 11.0)
Scanners per million population	6.60	10.48	6.8 * (5.1, 8.5)
Slovakia			
Exams per scanner	4932.1	6916.8	2.7 * (0.1, 5.7)
Exams per 1000 population	34.7	68.4	8.4 * (7.3, 9.8)
Scanners per million population	7.04	9.89	5.0 * (2.3, 7.9)
Spain			
Exams per scanner	4580.2	4646.4	1.5 * (0.6, 2.4)
Exams per 1000 population	63.0	84.7	4.3 * (3.4, 5.3)
Scanners per million population	13.76	18.22	2.9 * (2.4, 3.4)
Australia			
Exams per scanner	4304.5	3460.1	−4.1 * (−5.8, −0.6)
Exams per 1000 population	24.1	51.2	9.8 * (8.8, 11.2)
Scanners per million population	5.60	14.79	15.3 * (12.4, 19.0)

* The AAPC obtained from segmented regression analysis is significantly different from zero. ^†^ 2012 for the United States and Greece, 2013 for Italy, and 2014 for Poland. ^‡^ 2018 for Finland, and 2019 for France. Abbreviations: OECD, Organisation for Economic Co-operation and Development; AAPC, average annual per cent change; CI, confidence interval.

As showed in the time series in Figure 2, the number of MRI exams and equipment units varied greatly across OECD countries during the ten-year study period between 2011 and 2020. In 2020, or the last available year (Table 2), exams per 1000 population ranged from 40.6 in Poland to 122.8 in France, equipment units per million population ranged from 5.53 in Israel to 34.66 in the United States, and exams per equipment unit ranged from 1384.7 in Greece to 8425.5 in Israel.

Between 2011 and 2020, the United States was the only country in the sample that did not experience any significant increase or decrease in MRI exams and equipment units, which resulted in a nonsignificant trend in exams per equipment unit (AAPC = −0.5, 95% CI = −2.3 to +1.2). Greece and Italy registered a significant increase only in MRI equipment units (Greece: AAPC = +5.9, 95% CI = +5.2 to +6.7; Italy: AAPC = +2.7, 95% CI = +1.6 to +3.9) that resulted in a nonsignificant trend in exams per equipment unit for Greece (AAPC = −2.5, 95% CI = −8.8 to +4.8) and a significant decrease in exams per equipment unit for Italy (AAPC = −4.5, 95% CI = −7.4 to −1.7) (Table 2).

In the other 13 countries (Israel, South Korea, Belgium, Czechia, Finland, France, Lithuania, Luxembourg, the Netherlands, Poland, Slovakia, Spain, Australia), both MRI exams and equipment units significantly grew in number between 2011 and 2020. The trend in the exam-to-scanner ratio was statistically nonsignificant in Israel (AAPC = +0.5, 95% CI = −2.1 to +3.8), suggesting a similar increase in MRI exams and equipment units, while in South Korea, Belgium, Finland, Lithuania, the Netherlands, Poland, Slovakia, and Spain, the exam-to-scanner ratio increased significantly, suggesting that MRI exams grew at a faster pace than MRI equipment units. Lastly, in Czechia, France, Luxembourg, and Australia, the exam-to-scanner ratio decreased significantly, suggesting that MRI exams grew at a slower pace than MRI equipment units (Table 2).

Contrary to the main analysis, when 2020 was removed from the study period, we found that before the COVID-19 outbreak, the US experienced a significant increase in both MRI exams and equipment units (exams: AAPC = +2.5, 95% CI = +0.6 to +4.5; equipment units: AAPC = +1.8, 95% CI = +0.3 to +3.5) and that Greece experienced a significant increase in MRI exams (AAPC = +5.0, 95% CI = +3.8 to +6.8) (Table S2 in the Supplementary Materials).

Fixed-effects regression analysis revealed that, across all countries, a one-unit increase in CT exams per 1000 population over a year corresponded to a significant increase of 0.36 MRI exams per 1000 population (95% CI = +0.28 to +0.44, *p*-value < 0.001). On the contrary, no significant association was found for scanners per million population (*b* = +0.19, 95% CI = −0.03 to +0.41, *p*-value = 0.087).

#### 3.1.3. Positron Emission Tomography

**Figure 3:**
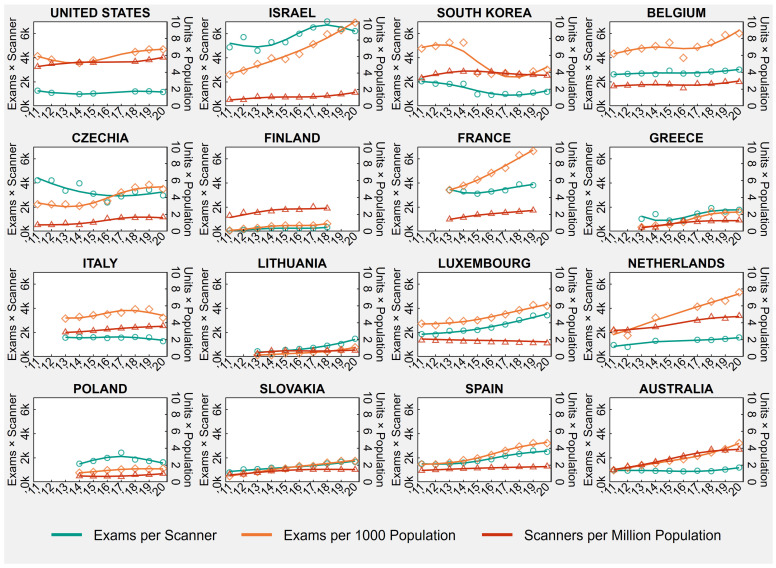
Positron Emission Tomography (PET) Exams and Scanners in 16 OECD Countries, Years 2011–2020.

Notes: Overall rates observed in all country-years are displayed in the background of each plot, below population-averaged cubic splines. In South Korea, the decrease in PET exams in 2015 is due to a change in payment standard for medical expenses. In Belgium, before 2016, PET activity was overestimated due to the partial inclusion of gamma camera activity. In France, before 2013, examinations practiced in private hospitals were wrongly compiled in ambulatory care, while during 2015, the official sources of data have been enhanced concerning all the equipment actually in use. In Poland, there was a change in data source in 2019 that led to the inclusion of specialty hospitals. In Australia, data relate to services rendered on a “fee-for-service” basis for which Medicare benefits were paid, which means that services that do not attract a Medicare benefit, such as those to public patients in hospitals or patients attending public Accident and Emergency Departments and public Outpatient Clinics, are excluded. Lastly, 2020 values for Luxembourg, the Netherlands, and Spain are provisional.

Abbreviations: OECD, Organisation for Economic Co-operation and Development.

Table 3 shows the country-specific number of PET exams per 1000 population and PET scanners per million population in the first and last available years between 2011 and 2020, as well the number of exams per scanner, obtained as ratio of the previously mentioned indicators. Table 3 also presents the country-specific AAPCs, while Figure 3 shows the trends of annual PET rates and ratios from 2011 to 2020.

As showed in the time series in Figure 3, the number of PET exams and scanners varied greatly across OECD countries during the ten-year study period between 2011 and 2020. In 2020 or the last available year (Table 3), exams per 1000 population ranged from 0.9 in Finland to 10.1 in Israel, scanners per million population ranged from 0.72 in Lithuania to 5.75 in the United States, and exams per scanner ranged from 333.6 in Finland to 6220.0 in Israel.

**Table 3:** Positron Emission Tomography (PET) Exams and Scanners in 16 OECD Countries, Years 2011 and 2020.

	2011 or First	2020 or Last	AAPC
	Available Year ^†^	Available Year ^‡^	(95% CI)
United States			
Exams per scanner	1278.4	1165.5	0.6 (−2.5, 4.2)
Exams per 1000 population	5.9	6.7	1.9 * (1.5, 2.6)
Scanners per million population	4.65	5.75	1.8 * (0.5, 3.1)
Israel			
Exams per scanner	4871.7	6220.0	3.6 * (0.7, 7.3)
Exams per 1000 population	3.8	10.1	11.2 * (9.8, 13.1)
Scanners per million population	0.77	1.63	7.3 * (3.9, 11.3)
South Korea			
Exams per scanner	2051.9	1180.9	−8.3 * (−14.4, −3.2)
Exams per 1000 population	6.8	4.3	−8.1 * (−15.1, −2.2)
Scanners per million population	3.30	3.61	0.9 * (0.3, 1.4)
Belgium			
Exams per scanner	2628.2	3019.7	1.3 * (0.6, 1.9)
Exams per 1000 population	6.2	8.6	3.1 * (0.9, 5.6)
Scanners per million population	2.36	2.86	1.7 (−0.1, 3.7)
Czechia			
Exams per scanner	4226.5	2974.7	−3.1 * (−5.5, −0.8)
Exams per 1000 population	3.2	5.0	7.2 * (2.5, 12.9)
Scanners per million population	0.76	1.68	10.8 * (7.2, 15.4)
Finland			
Exams per scanner	70.4	333.6	20.8 * (15.9, 31.2)
Exams per 1000 population	0.1	0.9	27.0 * (21.6, 36.4)
Scanners per million population	1.86	2.72	5.7 * (2.4, 9.0)
France			
Exams per scanner	3413.9	3818.6	2.3 * (1.8, 2.9)
Exams per 1000 population	4.9	9.5	12.5 * (11.9, 12.9)
Scanners per million population	1.43	2.48	9.8 * (9.5, 10.2)
Greece			
Exams per scanner	1040.0	1787.0	10.5 * (2.7, 22.7)
Exams per 1000 population	0.5	2.3	24.9 * (21.9, 30.9)
Scanners per million population	0.46	1.31	17.2 * (14.6, 20.4)
Italy			
Exams per scanner	1567.5	1277.6	−1.4 (−3.4, 0.6)
Exams per 1000 population	4.5	4.6	1.3 (−0.4, 3.5)
Scanners per million population	2.89	3.63	3.6 * (3.0, 4.2)
Lithuania			
Exams per scanner	445.0	1479.5	22.2 * (18.0, 28.0)
Exams per 1000 population	0.2	1.1	26.6 * (22.5, 32.1)
Scanners per million population	0.34	0.72	4.0 * (0.5, 7.7)
Luxembourg			
Exams per scanner	1862.0	3423.0	7.7 * (6.7, 8.9)
Exams per 1000 population	3.9	6.0	5.9 * (5.2, 6.7)
Scanners per million population	1.93	1.59	−1.8 * (−2.3, −1.3)
Netherlands			
Exams per scanner	960.6	1588.2	6.1 * (4.0, 8.8)
Exams per 1000 population	3.0	7.6	11.8 * (9.3, 15.4)
Scanners per million population	3.12	4.82	5.5 * (4.3, 6.9)
Poland			
Exams per scanner	1520.6	1636.8	0.3 (−2.0, 3.0)
Exams per 1000 population	1.1	1.6	6.2 * (5.9, 6.4)
Scanners per million population	0.71	1.00	6.2 * (3.0, 9.3)
Slovakia			
Exams per scanner	806.0	1676.9	7.4 * (5.8, 9.6)
Exams per 1000 population	0.7	2.5	15.1 * (13.8, 17.3)
Scanners per million population	0.93	1.47	6.6 * (6.3, 6.9)
Spain			
Exams per scanner	1526.1	2500.1	7.8 * (6.1, 9.9)
Exams per 1000 population	2.1	4.6	10.7 * (8.8, 13.2)
Scanners per million population	1.35	1.86	3.3 * (3.0, 3.6)
Australia			
Exams per scanner	983.0	1201.1	2.5 * (2.0, 2.8)
Exams per 1000 population	1.4	4.6	13.0 * (12.1, 14.3)
Scanners per million population	1.43	3.85	10.4 * (9.6, 11.6)

* The AAPC obtained from segmented regression analysis is significantly different from zero. ^†^ 2013 for France, Greece, Italy, and Lithuania, and 2014 for Poland. ^‡^ 2018 for Finland, and 2019 for France. Abbreviations: OECD, Organisation for Economic Co-operation and Development; AAPC, average annual per cent change; CI, confidence interval.

Between 2011 and 2020, Belgium registered a significant increase only in PET exams (AAPC = +3.1, 95% CI = +0.9 to +5.9) that resulted in a significant increase in exams per scanner (AAPC = +1.3, 95% CI = +0.6 to +1.9), while Italy registered a significant increase only in PET scanners (AAPC = +3.6, 95% CI = +3.0 to +4.2) that resulted in a nonsignificant trend in exams per scanner (AAPC = −1.4, 95% CI = −3.4 to +0.6). Luxembourg experienced a significant increase in PET exams (AAPC = +5.9, 95% CI = +5.2 to +6.7) in conjunction with a significant decrease in the number of PET scanners (AAPC = −1.8, 95% CI = −2.3 to −1.3), which translated into a significant increase in exams per scanner (AAPC = +7.7, 95% CI = +6.7 to +8.9). On the contrary, South Korea experienced a significant decrease in PET exams (AAPC = −8.1, 95% CI = −15.1 to −2.2) in conjunction with a significant increase in the number of PET scanners (AAPC = +0.9, 95% CI = +0.3 to +1.4), which translated into a significant decrease in exams per scanner (AAPC = −8.3, 95% CI = −14.4 to −3.2) (Table 3). In the other 12 countries (the United States, Israel, Czechia, Finland, France, Greece, Lithuania, the Netherlands, Poland, Slovakia, Spain, Australia), both PET exams and scanners significantly grew in number between 2011 and 2020. The trend in the exam-to-scanner ratio was statistically nonsignificant in the United States and Poland (United States: AAPC = +0.6, 95% CI = −2.5 to +4.2; Poland: AAPC = +0.3, 95% CI = −2.0 to +3.0). This suggests that in these countries, PET exams and scanners increased at a similar pace. In Israel, Finland, France, Greece, Lithuania, the Netherlands, Slovakia, Spain, and Australia, the exam-to-scanner ratio increased significantly, suggesting that PET exams grew at a faster pace than scanners. Lastly, in Czechia, the exam-to-scanner ratio decreased significantly (AAPC = −3.1, 95% CI = −5.5 to −0.8), suggesting that PET exams grew at a slower pace than PET scanners (Table 3).

Contrary to the main analysis, when 2020 was removed from the study period, we found that Italy experienced a significant increase in the number of PET exams before the outbreak of COVID-19 (AAPC = +4.0, 95% CI = +3.5 to +4.3) (Table S3 in the Supplementary Materials).

## 4. Discussion

This study evaluates the trends of exam-to-diagnostic technology ratio through the trend analysis of the number of examinations per population and the number of CT/PET scanners or MRI equipment units per population in the 2011–2020 period for 16 OECD countries. Four distinct behaviors can be identified:

1. The ratio of exams to diagnostic technologies decreases with an increase in the number of diagnostic technologies per population and a reduction in the number of examinations per population;
2. The ratio decreases with a greater increase in diagnostic technologies per population compared to an increase in the number of examinations per population;
3. The ratio increases due to a smaller increase in diagnostic technologies per population compared to an increase in the number of examinations per population;
4. The ratio increases with a reduction in diagnostic technologies per population and an increase in the number of examinations per population.

Behaviors #1 and #2 suggest an excess of supply, which results in inefficient use of diagnostic technologies. Behavior #4, which has a reduced supply, suggests a better exploitation of the production capacity of diagnostic technologies. Finally, behavior #3 is difficult to interpret. Indeed, not having a benchmark for the ideal number of exams per equipment unit makes it difficult to make a judgment on the actual need to increase the number of diagnostic technologies, or whether it would be sufficient to optimize the use of existing technologies. Behaviors #1 and #2, which show a more rapid stockpiling of devices, may be affected by multiple factors. A likely factor may be represented by significant advancements in imaging technology. While older generations of scanners may still be available, their usage may have declined due to the increased efficacy of advanced machines. The extent to which new equipment substitutes for other technologies and the scarce decommissioning of older diagnostic technology devices can lead to over-supply.

Another possible cause may be associated with disputable evaluation of financial investments that, correctly, should incorporate both the benefits and costs of new technologies. In this regard, although there is no official guidance on the provision of imaging devices (CT, MRI, and PET), setting priorities and recommendations for such technologies would be a step towards achieving operational efficiency, which entails the accomplishment of the objectives with minimal costs. Accordingly, optimizing resource allocation and effectively exploiting the available resources contribute significantly to maximizing the benefits. Such recommendations seek to ensure that healthcare resources are fairly allocated [18]. In this respect, to successfully implement priority settings, recently, the International Network of Agencies for Health Technology Assessment (INAHTA) launched a list of top-ten challenges, including a few warnings about shifting political behavior, to encourage a prompt translation of HTA fundamentals into policy [19]. To support the validity of this position, this study shows a virtuous example in Luxembourg, where a reduction in PET and CT scanners was possible despite an increasing demand for radiology examinations. It is reasonable to assume the adopted assessment and regulation of medical equipment as an explanation for this gain in efficiency. It is a prerogative of the Ministry of Health and Permanent Hospital Commission to promote HTA strategies for planning and managing with a politically centralized approach; therefore, the Luxembourgian Government fostered a cost-effective HTA plan in collaboration with health insurance agencies [20].

The analysis of the trend in the demand for diagnostic tests and in the offer of diagnostic technologies shows that these indicators do not always have the same slope or direction. This interesting result raises questions about the ability of supplying to condition the demand for diagnostic services. For example, in the case of behavior #2, where there is an increase in diagnostic technologies that exceeds the demand for diagnostic tests, it seems that supply does not have a great influence on demand. In behavior #1, demand seems to decline with an increase in supply, and in behavior #4, demand increases with a decrease in supply. Certainly, more targeted research to investigate the elasticity of demand with respect to supply is needed. Additionally, since demand in this study is measured as the number of diagnostic tests performed, it is possible that there is still an influence of supply on the demand for diagnostic tests not yet performed, resulting in a waiting list. Moreover, it is certainly possible that part of the diagnostic tests delivered is already the result of overutilization or inappropriate utilization conditioned by the supply. However, the different slopes and trend directions suggest the role of other significant factors in determining the demand for diagnostic exams. For instance, demographic and epidemiological variations over the decades could have notably impacted the level of assistance required, including diagnostic imaging. In addition, the different increase over a year in CT exams compared to MRI exams resulting from fixed-effects regression reflects basic and substantial diversity between these procedures. First, CT has several advantages over MRI, which is not feasible in daily clinical practice and cannot provide for requested information. In general, CT devices are more easily available and CT examinations are quick to perform, being the technique of choice for cancer follow-up, and for traumatic and non-traumatic emergencies. Indeed, the presence of metallic implants or foreign bodies and common medical devices as pacemakers represent an absolute contraindication for MRI, as well as claustrophobia and anxiety that make such procedure unsustainable for many patients due to uncomfortable closed scanner bore and loud noise. The duration of MRI exams, which can last up to 30–40 min, can also lead to decreased imaging quality due to patients’ movements. Furthermore, MRI equipment units tend to be more expensive than CT scans. This is primarily because MRI technology is more complex and expensive to maintain and operate [21].

A secondary, but not less relevant, finding of our study showed that until 2019, the pre-COVID-19 period, all countries recorded an increase in the number of exams per 1000 population. Only Italy saw a reduction in the number of MRI exams. Various factors contributed to this phenomenon, such as advances in imaging technology, aging populations, epidemiological transition, and healthcare system characteristics, such as the payment system. Also work organization and availability of doctors and other health workers in a sufficient number can have affected the number of exams. Indeed, it is plausible that insufficient recruitment of new personnel may have further exacerbated the workload of radiologists due to excessive number of patients per scanner. Work overload can lead to decreased efficiency, further intensifying the need for rushed or inaccurate examinations. This creates a detrimental cycle of inefficient workflow. Additionally, specialists’ behavior and education can also have driven the prescription of radiology exams. Studies have highlighted that reassuring patients is a leading reason for prescription, sometimes inducing professionals to practice defensive medicine [22,23,24]. Lastly, the social context may have driven the culture of overuse regarding health and healthcare issues. This suggestion gains some support by critical perspective from Canada, under which the increasing demand for diagnostic tests seems to be the consequence of a multifaceted context that shapes the health beliefs, values, and behaviors of both patients and providers [25].

In this field, the “patient-centered care” is a novel reform in the US that aims to engage patients in managing their own health and forge partnerships with their clinicians by obtaining real-time access to their own medical records and science-based comparative effectiveness information for a better personalization of medical care. In this context, the Choosing Wisely campaign was fostered by medical specialty societies, the American Board of Internal Medicine (ABIM) Foundation, and Consumer Reports (a nonprofit consumer organization), by selecting and listing five tests, treatments, or services to reconsider clinical usefulness and validity from both patients’ and clinicians’ points of view [26].

In conclusion, studies on the determinants of inefficient use and oversupply of imaging technologies are currently lacking. Hence, future analysis should focus on understanding the oversupply of devices without any evident and appropriate growth of demand or the underutilization of currently available radiology devices.

Both professionals and policy-makers must be involved in the application of HTA strategies to manage the appropriate allocation of economic, personnel, and technology resources. Managers must be involved in a more efficient use of technologies. These issues are particularly relevant because the aging population strongly affects economic, social, and healthcare systems by modifying health needs in terms of integrated person-centered care and long-term care. In recent decades, the proportion of people older than 65 years has significantly increased from less than 9% in 1960, and forecasts predict that this trend will continue. Indeed, projections highlight a growth from 17.3% in 2019 to 26.7% by 2050 across OECD countries, with even a few countries expected to see over one-third their population aged 65 and older and an acute increase in the number of people aged 80 and over. In Italy, this trend is even worse, where the population aged 65 will exceed one-third by 2050 and one in eight people will be 80 years old and over. Exceptional longevity of the population will likely lead to exceptional levels of both acute and chronic morbidities, requiring exceptional intensity of health assistance. Similarly, chronic diseases will challenge the traditional social welfare state and resource management. Cancer is showing improved survival rates in countries with high social settings [9,27,28]. The implications of this include the need to assess emerging novel health needs such as greater demand for medical examinations and prescriptions.

The main weakness of this study is that the analysis was conducted at the national level, and the state-wide distribution was not analyzed. An analysis carried out within the national context may highlight an uneven distribution of diagnostic technologies that could represent an excess of supply in certain geographical areas or lack of instrumentation in others, which may result in barriers to optimal utilization. Recently, a German study revealed a strong variation in imaging demand and use of PET and CT imaging units, partly due to regional variations in disease burden and supply factors primarily in ambulatory settings [29].

Similarly, aggregated data inevitably conceal large within-country variations in sociodemographic and epidemiological variables or other factors, such as the number of radiologists, which are able to play a role in the use of diagnostic technologies or in the efficiency of use of diagnostic technologies. A pilot study into the presence of radiologists and the implementation of PET-CT imaging stewardship resulted in a reduced volume of examinations due to monitoring low-value indications and appropriateness of imaging requests from clinicians through radiology consultation [30]. In addition, our analysis was performed at the country level, and countries with different financial and organizational factors of healthcare systems were both considered. Several previous studies have observed that the healthcare system or insurance system may affect the use or overuse of diagnostic exams [31,32].

## 5. Conclusions

Efficient use of diagnostic technologies accounts for the capacity utilization of devices and balanced supply and demand for population health needs. In this field, decision-making requires the involvement of multiple stakeholders, suggesting a multifaceted approach for better allocation of professional, technological, and economic resources. Considering the evolving needs of the population, and the related increasing demand for diagnostics, proper investments and novel strategies in the management of technologies are necessary to guarantee operational and allocative efficiency.

## Data Availability

Original data are available at the following link: https://stats.oecd.org/.

## Supplementary Materials

The following supporting information can be downloaded at: https://www.mdpi.com/article/10.3390/healthcare11142078/s1, Table S1: Computed Tomography (CT) Exams and Scanners in 16 OECD Countries, Years 2011–2019; Table S2: Magnetic Resonance Imaging (MRI) Exams and Units in 16 OECD Countries, Years 2011–2019; Table S3: Positron Emission Tomography (PET) Exams and Scanners in 16 OECD Countries, Years 2011–2019.
