# Supplementary material for: Diagnostic Technology: Trends of Use and Availability in a 10-Year Period (2011–2020) among Sixteen OECD Countries
Source: Healthcare (Basel). 2023 Jul 20;11(14):2078. doi: 10.3390/healthcare11142078 (PMC10378781; doi:10.3390/healthcare11142078)
Supplement: Supplementary file 1 [file healthcare-11-02078-s001.zip › healthcare-2445248-supplementary.pdf]

Supplementary tables

Table S1. Computed Tomography (CT) Exams and Scanners in 16 OECD Countries, Years 2011–2019.

	Year									AAPC
	2011	2012	2013	2014	2015	2016	2017	2018	2019	(95% CI)
United States										
Exams per scanner	6695.4	5851.2	5529.3	6215.1	5984.8	6062.8	5997.1	6094.1	6196.6	−0.1 (−1.5, 1.4)
Exams per 1000 population	273.8	256.8	240.5	255.0	245.4	253.8	256.2	271.4	278.4	0.0 (−0.7, 1.3)
Scanners per million population	40.89	43.89	43.49	41.04	41.00	41.86	42.72	44.53	44.92	0.6 (−1.3, 2.6)
Israel										
Exams per scanner	14668.7	15720.7	15816.3	15000.1	13924.3	14375.3	15065.0	14633.1	15988.5	0.0 (−1.7, 1.8)
Exams per 1000 population	126.6	139.1	141.3	140.6	131.3	136.3	145.2	146.6	158.9	1.8 (−0.6, 4.5)
Scanners per million population	8.63	8.85	8.93	9.37	9.43	9.48	9.64	10.02	9.94	1.9* (1.5, 2.2)
South Korea										
Exams per scanner	3660.1	3869.5	4016.0	4446.6	4721.6	5058.5	5360.2	5916.9	6278.5	7.2* (6.8, 7.7)
Exams per 1000 population	131.0	142.9	150.6	163.9	174.8	191.3	205	228.4	248.5	8.3* (8.0, 8.6)
Scanners per million population	35.79	36.93	37.50	36.85	37.03	37.82	38.24	38.60	39.58	1.0* (0.5, 1.5)
Belgium										
Exams per scanner	··	··	8120.0	8868.6	8411.1	8348.9	8417.8	8451.2	8497.1	−0.1 (−0.9, 0.8)
Exams per 1000 population	··	··	186.3	193.1	198.4	199.7	200.5	201.9	204.9	1.5* (1.3, 1.7)
Scanners per million population	··	··	22.94	21.77	23.59	23.92	23.82	23.89	24.11	1.4* (0.5, 2.2)
Czechia										
Exams per scanner	6061.7	6285.9	6374.4	6504.3	6321.8	6922.5	6569.2	6891.4	6958.8	1.5* (1.1, 2.0)
Exams per 1000 population	89.5	94.5	95.8	98.3	101.9	107.4	103.5	110.9	114.1	2.8* (2.4, 3.2)
Scanners per million population	14.77	15.03	15.03	15.11	16.12	15.52	15.76	16.09	16.40	1.2* (0.9, 1.5)
Finland										
Exams per scanner	1308.8	1603.3	1807.2	1825.1	1814.8	1723.1	2204.4	3484.8	··	11.8* (5.3, 20.4)
Exams per 1000 population	27.9	34.9	39.2	39.1	39.1	41.7	54.0	57.5	··	9.3* (5.3, 14.2)
Scanners per million population	21.34	21.80	21.70	21.42	21.53	24.20	24.51	16.50	··	−0.1 (−3.8, 3.7)

France										
Exams per scanner	12338.3	12753.4	12713.1	11709.3	11385.1	11446.3	10927.3	11069.8	10953.1	−2.1* (−3.3, −0.9)
Exams per 1000 population	154.6	172.1	184.2	179.4	188.7	194.0	189.6	195.2	198.6	2.9* (2.1, 3.7)
Scanners per million population	12.53	13.49	14.49	15.32	16.57	16.95	17.35	17.63	18.13	4.7* (4.6, 4.8)
Greece										
Exams per scanner	··	5420.1	4173.4	4854.1	4040.9	4186.0	5685.3	5265.3	4595.0	1.3 (−2.7, 5.5)
Exams per 1000 population	··	181.1	140.4	168.0	146.0	150.3	194.5	213.9	195.4	4.7 (−1.0, 10.7)
Scanners per million population	··	33.41	33.65	34.61	36.13	35.91	34.22	40.62	42.53	3.7* (2.2, 4.5)
Italy										
Exams per scanner	··	··	2605.6	2661.4	2655.1	2501.6	2599.8	2662.6	2668.7	0.2 (−0.8, 1.1)
Exams per 1000 population	··	··	86.3	87.6	88.4	85.8	89.9	93.6	97.3	1.8* (1.1, 2.5)
Scanners per million population	··	··	33.10	32.90	33.31	34.29	34.57	35.15	36.45	1.7* (1.0, 2.3)
Lithuania										
Exams per scanner	3555.5	3255.2	3683.5	3997.7	4512.7	4488.1	4556.5	4980.9	5004.7	5.6* (4.3, 6.9)
Exams per 1000 population	71.6	77.4	87.2	88.6	94.8	103.3	106.3	120.9	132.5	7.5* (6.5, 8.6)
Scanners per million population	20.14	23.76	23.67	22.17	21.00	23.01	23.33	24.27	26.48	1.8 (−0.2, 3.9)
Luxembourg										
Exams per scanner	7493.3	7919.6	8614.7	8802.8	10706.1	11154.5	11404.5	11988.7	12473.1	7.1* (6.0, 8.2)
Exams per 1000 population	202.1	209.0	206.0	206.9	206.6	211.8	212.8	219.6	224.1	1.0* (0.8, 1.2)
Scanners per million population	25.08	24.48	22.08	21.57	17.56	17.14	16.77	16.45	16.13	−5.4* (−6.3, −4.7)
Netherlands										
Exams per scanner	5654.5	··	··	5962.6	5875.0	6801.1	7149.7	7403.4	7456.7	4.2* (2.8, 6.0)
Exams per 1000 population	70.8	··	··	79.5	80.8	88.7	96.4	105.3	110.9	5.9* (5.7, 6.2)
Scanners per million population	12.52	··	··	13.34	13.75	13.04	13.48	14.22	14.87	1.8* (0.9, 2.8)
Poland										
Exams per scanner	··	··	··	4208.9	4087.3	4404.6	4825.8	4707.4	5536.3	5.6* (3.2, 7.8)
Exams per 1000 population	··	··	··	65.8	70.2	76.3	81.5	85.4	100.6	8.1* (7.0, 9.0)
Scanners per million population	··	··	··	15.63	17.16	17.33	16.88	18.14	18.17	2.4* (0.4, 4.3)
Slovakia										
Exams per scanner	6602.3	6924.5	8006.6	7770.1	8734.1	9375.2	8905.4	8450.9	9004.8	3.2* (2.1, 4.3)
Exams per 1000 population	99.1	107.6	122.8	134.8	156.2	162.3	153.9	155.2	160.1	6.3* (5.0, 8.0)

Scanners per million population	15.00	15.53	15.33	17.35	17.88	17.31	17.28	18.36	17.78	2.4* (1.4, 3.4)
Spain										
Exams per scanner	5298.1	5260.7	5464.7	5757.1	5819.3	5990.2	6163.1	6175.4	6475.3	2.6* (2.4, 2.9)
Exams per 1000 population	88.2	90.4	96.1	101.3	104.9	109.7	114.9	118.1	124.3	4.4* (4.2, 4.7)
Scanners per million population	16.64	17.19	17.59	17.60	18.02	18.31	18.65	19.12	19.2	1.8* (1.4, 2.1)
Australia										
Exams per scanner	2057.7	2060.6	2047.2	2058.6	2007.0	1894.0	1958.6	2000.5	2020.2	−0.5 (−1.3, 0.5)
Exams per 1000 population	91.2	104.1	109.8	115.4	119.5	119.3	126.0	134.6	140.9	4.7* (3.5, 6.0)
Scanners per million population	44.32	50.5	53.66	56.06	59.54	63.00	64.34	67.29	69.74	5.7* (5.2, 6.3)

Notes: Cells have two mid-dots (··) if no data are available; * indicates that the AAPC obtained from segmented regression analysis is significantly different from zero. In France, before 2013 examinations practiced in private hospitals were wrongly compiled in ambulatory care, while during 2015 the official sources of data have been enhanced concerning all the equipment actually in use. In Poland, there was a change in data source in 2019 that led to the inclusion of specialty hospitals. In Australia, data relate to services rendered on a “fee-for-service” basis for which Medicare benefits were paid, which means that services that do not attract a Medicare benefit, such as those to public patients in hospitals or patients attending public Accident and Emergency Departments and public Outpatient Clinics, are excluded.
Abbreviations: OECD, Organisation for Economic Co-operation and Development; AAPC, average annual per cent change; CI, confidence interval.

Table S2. Magnetic Resonance Imaging (MRI) Exams and Units in 16 OECD Countries, Years 2011–2019.

	Year									AAPC
	2011	2012	2013	2014	2015	2016	2017	2018	2019	(95% CI)
United States										
Exams per scanner	··	3042.1	3012.5	2876.0	3020.4	3287.0	2950.6	3042.1	3163.8	0.6 (−0.5, 1.7)
Exams per 1000 population	··	104.8	106.9	109.6	117.9	120.7	111.0	119.3	127.9	2.5* (0.6, 4.5)
Scanners per million population	··	34.46	35.50	38.11	39.02	36.73	37.63	39.22	40.43	1.8* (0.3, 3.5)
Israel										
Exams per scanner	7276.2	8530.8	9446.8	8234.5	8334.0	6915.6	7117.1	8621.3	9146.2	0.6 (−3.5, 5.8)
Exams per 1000 population	19.7	28.0	30.5	32.1	34.8	36.4	38.4	46.6	47.5	9.2* (6.8, 12.1)
Scanners per million population	2.70	3.29	3.23	3.89	4.18	5.27	5.39	5.40	5.19	8.4* (7.1, 9.9)
South Korea										
Exams per scanner	1095.6	1036.9	1074.9	1173.7	1187.5	1216.1	1247.7	1629.7	2308.3	10.1* (9.2, 11.2)
Exams per 1000 population	23.3	24.2	26.2	29.9	31.2	33.8	36.3	49.1	73.8	15.4* (14.7, 16.1)
Scanners per million population	21.27	23.37	24.35	25.50	26.27	27.82	29.13	30.11	31.99	4.7* (4.3, 5.2)
Belgium										
Exams per scanner	6564.6	6968.2	7114.2	6919.8	7307.6	7674.6	8095.4	8196.5	8540.1	3.2* (2.6, 3.9)
Exams per 1000 population	70.2	74.0	77.1	81.5	85.6	89.4	93.9	95.4	98.1	4.2* (4.1, 4.3)
Scanners per million population	10.69	10.62	10.84	11.78	11.71	11.65	11.60	11.64	11.49	1.2* (0.7, 1.7)
Czechia										
Exams per scanner	5678.8	6221.9	6095.5	6249.2	5747.6	5846.8	5582.4	5288.3	5777.5	−1.6* (−2.7, −0.5)
Exams per 1000 population	39.0	43.2	45.2	46.3	48.0	49.8	52.7	54.7	60.1	4.8* (3.9, 5.6)
Scanners per million population	6.86	6.95	7.42	7.41	8.34	8.52	9.44	10.35	10.40	6.0* (5.6, 6.4)
Finland										
Exams per scanner	1318.2	1447.2	1549.0	1520.9	1609.2	1628.9	1736.7	1809.6	··	4.0* (3.0, 5.0)
Exams per 1000 population	26.7	31.3	34.2	35.4	41.7	41.5	47.0	49.5	··	8.5* (7.6, 9.5)
Scanners per million population	20.23	21.61	22.06	23.25	25.91	25.48	27.05	27.38	··	4.6* (3.9, 5.3)
France										
Exams per scanner	8991.9	9477.8	9506.1	8650.3	8170.6	8173.2	8031.3	8095.0	8004.4	−2.2* (−3.5, −0.6)
Exams per 1000 population	67.5	82.0	89.3	93.9	102.6	110.7	114.0	119.2	122.8	7.8* (5.6, 10.0)

Scanners per million population	7.51	8.65	9.40	10.86	12.56	13.55	14.20	14.73	15.34	9.2* (8.8, 9.5)
Greece										
Exams per scanner	··	3099.2	2438.0	2550.2	2444.0	2379.3	2761.2	2841.1	2739.6	−0.4 (−2.0, 2.2)
Exams per 1000 population	··	67.9	53.8	58.3	60.5	64.0	73.2	83.4	87.4	5.0* (3.8, 6.8)
Scanners per million population	··	21.91	22.07	22.86	24.77	26.91	26.50	29.35	31.90	5.7* (4.4, 7.1)
Italy										
Exams per scanner	··	··	3107.7	3034.1	2760.7	2375.4	2490.0	2569.7	2495.1	−3.9* (−5.7, −2.2)
Exams per 1000 population	··	··	78.3	79.5	78.0	67.5	71.4	73.7	75.4	−1.4 (−3.6, 0.8)
Scanners per million population	··	··	25.20	26.19	28.24	28.40	28.66	28.68	30.22	2.8* (2.3, 3.2)
Lithuania										
Exams per scanner	4040.4	2760.6	3196.1	3461.7	3702.0	3692.5	4047.7	4602.0	4798.6	5.9* (3.3, 8.7)
Exams per 1000 population	24.0	27.7	33.5	36.6	40.8	45.1	50.1	57.5	67.0	13.0* (11.6, 14.8)
Scanners per million population	5.94	10.04	10.48	10.57	11.02	12.2	12.37	12.49	13.96	6.3* (3.4, 9.6)
Luxembourg										
Exams per scanner	5681.7	5653.1	5907.7	5996.6	6064.3	6306.0	6231.4	6509.3	5604.9	0.7 (−0.1, 1.3)
Exams per 1000 population	82.5	80.3	82.4	82.2	81.9	83.8	81.4	83.5	90.6	1.1* (0.1, 1.7)
Scanners per million population	13.50	13.18	12.88	12.58	12.29	12.00	11.74	11.51	14.52	0.5 (−0.7, 1.1)
Netherlands										
Exams per scanner	3880.9	··	··	3982.2	4136.2	3815.5	3925.8	3994.0	4297.3	0.7 (−0.5, 1.9)
Exams per 1000 population	50.0	··	··	51.2	51.8	48.8	51.1	52.2	59.5	1.6* (0.5, 2.3)
Scanners per million population	12.88	··	··	12.87	12.51	12.80	13.02	13.06	13.84	0.7* (0.3, 1.0)
Poland										
Exams per scanner	··	··	··	3996.4	3644.2	3785.6	4349.0	4040.5	4552.3	3.5* (0.8, 6.4)
Exams per 1000 population	··	··	··	26.4	27.8	29.8	34.5	37.2	42.2	10.3* (8.3, 12.4)
Scanners per million population	··	··	··	6.60	7.63	7.87	7.93	9.22	9.27	6.5* (5.3, 7.6)
Slovakia										
Exams per scanner	4932.1	6499.0	6958.4	6212.5	6415.3	6808.1	6585.4	7281.7	7727.5	3.5* (1.4, 6.0)
Exams per 1000 population	34.7	40.9	46.3	51.6	56.8	61.4	63.0	69.5	73.7	9.7* (9.1, 10.6)
Scanners per million population	7.04	6.29	6.65	8.30	8.85	9.02	9.56	9.55	9.53	5.5* (3.6, 7.6)
Spain										
Exams per scanner	4580.2	4369.1	4533.3	4989.9	4942.9	5172.6	5407.4	5370.7	5621.0	3.1* (2.0, 4.3)

Exams per 1000 population	63.0	64.5	69.5	77.4	78.3	83.2	88.6	92.4	99.1	5.9* (5.5, 6.3)
Scanners per million population	13.76	14.77	15.34	15.51	15.85	16.09	16.38	17.20	17.63	2.8* (2.3, 3.3)
Australia										
Exams per scanner	4304.5	4727.5	1994.0	2409.1	2824.1	2964.1	3164.6	3413.8	3471.6	−5.1* (−7.1, −2.4)
Exams per 1000 population	24.1	26.0	27.6	35.3	40.9	42.4	44.8	48.1	51.3	10.6* (9.3, 12.4)
Scanners per million population	5.60	5.50	13.84	14.65	14.49	14.30	14.15	14.09	14.78	17.3* (13.7, 21.6)

Notes: Cells have two mid-dots (··) if no data are available; * indicates that the AAPC obtained from segmented regression analysis is significantly different from zero. In Israel, the increase in the number of MRI exams in 2018 is mainly due to an increasing number of hospitals reporting these data. In Belgium, since 2016 data are based on the national registry for devices of medical image and correspond to the number of MRI-devices. In France, before 2013 examinations practiced in private hospitals were wrongly compiled in ambulatory care, while during 2015 the official sources of data have been enhanced concerning all the equipment actually in use. In Poland, there was a change in data source in 2019 that led to the inclusion of specialty hospitals. In Australia, data relate to services rendered on a “fee-for-service” basis for which Medicare benefits were paid, which means that services that do not attract a Medicare benefit, such as those to public patients in hospitals or patients attending public Accident and Emergency Departments and public Outpatient Clinics, are excluded.

Abbreviations: OECD, Organisation for Economic Co-operation and Development; AAPC, average annual per cent change; CI, confidence interval

Table S3. Positron Emission Tomography (PET) Exams and Scanners in 16 OECD Countries, Years 2011–2019.

	Year									AAPC
	2011	2012	2013	2014	2015	2016	2017	2018	2019	(95% CI)
United States										
Exams per scanner	1278.4	1098.0	··	988.9	1044.7	··	··	1216.3	1229.5	0.5 (−4.5, 6.3)
Exams per 1000 population	5.9	5.5	··	5.1	5.4	··	··	6.4	6.7	2.2 (−1.4, 6.3)
Scanners per million population	4.65	5.00	··	5.14	5.13	··	··	5.25	5.45	1.5* (0.3, 2.8)
Israel										
Exams per scanner	4871.7	5716.7	4596.4	5295.7	5293.3	5999.9	6520.7	7014.5	6393.7	4.9* (1.7, 8.7)
Exams per 1000 population	3.8	4.3	5.1	5.8	5.7	6.3	7.5	8.7	9.2	11.4* (9.2, 14.3)
Scanners per million population	0.77	0.76	1.12	1.10	1.07	1.05	1.15	1.24	1.44	6.3* (2.4, 10.8)
South Korea										
Exams per scanner	2051.9	1853.0	1831.5	1846.3	977.0	926.9	1002.6	983.9	1118.5	−10.2* (−16.9, −4.4)
Exams per 1000 population	6.8	7.1	7.5	7.5	3.9	3.8	3.9	3.7	4.1	−9.5* (−18.2, −1.9)
Scanners per million population	3.30	3.80	4.10	4.08	3.96	4.06	3.89	3.78	3.69	1.3* (0.5, 2.1)
Belgium										
Exams per scanner	2628.2	2670.5	2699.3	2644.7	2930.9	2695.1	2657.3	2864.1	2921.3	1.0 (−0.1, 2.1)
Exams per 1000 population	6.2	6.5	6.8	7.1	7.5	5.7	7.0	7.5	8.4	2.6 (−0.1, 5.5)
Scanners per million population	2.36	2.43	2.51	2.68	2.57	2.12	2.64	2.63	2.87	1.5 (−0.9, 4.0)
Czechia										
Exams per scanner	4226.5	4216.5	3366.6	3976.6	3115.5	2390.1	2891.9	3282.3	3435.7	−3.2* (−5.7, −0.9)
Exams per 1000 population	3.2	3.2	3.2	3.0	3.2	3.6	4.6	5.2	5.5	7.9* (6.6, 9.2)
Scanners per million population	0.76	0.76	0.95	0.76	1.04	1.51	1.60	1.60	1.59	12.0* (6.9, 19.1)
Finland										
Exams per scanner	70.4	118.7	179.2	265.8	257.6	269.4	252.4	333.6	··	20.8* (15.4, 26.4)
Exams per 1000 population	0.1	0.3	0.4	0.6	0.7	0.7	0.7	0.9	··	27.0* (21.1, 34.2)
Scanners per million population	1.86	2.22	2.21	2.38	2.55	2.55	2.90	2.72	··	5.7* (2.5, 8.8)
France										
Exams per scanner	··	··	3413.9	3305.5	3115.2	3300.8	3399.5	3889.6	3818.6	2.3* (1.8, 2.9)
Exams per 1000 population	··	··	4.9	5.4	6.1	6.9	7.5	9.0	9.5	12.5* (11.9, 12.9)

Scanners per million population	··	··	1.43	1.63	1.95	2.08	2.20	2.32	2.48	9.8* (9.5, 10.2)
Greece										
Exams per scanner	··	··	1040.0	1430.0	900.0	884.6	1468.2	1923.4	1639.7	12.5* (4.5, 23.2)
Exams per 1000 population	··	··	0.5	0.7	0.8	1.1	1.6	2.3	2.1	30.3* (23.7, 40.6)
Scanners per million population	··	··	0.46	0.46	0.92	1.21	1.12	1.21	1.31	19.6* (16.0, 23.9)
Italy										
Exams per scanner	··	··	1567.5	1620.9	1602.5	1538.5	1572.5	1622.7	1589.9	0.1 (−0.7, 0.8)
Exams per 1000 population	··	··	4.5	4.7	4.9	5.0	5.2	5.6	5.6	4.0* (3.5, 4.3)
Scanners per million population	··	··	2.89	2.90	3.05	3.23	3.34	3.44	3.55	3.9* (3.4, 4.3)
Lithuania										
Exams per scanner	··	··	445.0	311.5	558.5	636.5	726.5	902.5	1048.0	20.8* (17.4, 24.9)
Exams per 1000 population	··	··	0.2	0.2	0.4	0.4	0.5	0.6	0.8	25.0* (21.4, 29.0)
Scanners per million population	··	··	0.34	0.68	0.69	0.70	0.71	0.71	0.72	4.2 (−0.4, 9.1)
Luxembourg										
Exams per scanner	1862.0	1825.0	2133.0	2153.0	2205.0	2400.0	2660.0	3029.0	3395.0	8.1* (7.4, 8.7)
Exams per 1000 population	3.9	3.7	4.2	4.2	4.3	4.6	5.0	5.5	6.1	6.2* (5.6, 6.7)
Scanners per million population	1.93	1.88	1.84	1.80	1.76	1.71	1.68	1.64	1.61	−1.8* (−2.4, −1.1)
Netherlands										
Exams per scanner	960.6	799.6	··	1308.4	··	··	1390.4	1373.4	1461.8	6.3* (3.7, 8.9)
Exams per 1000 population	3.0	2.5	··	4.6	··	··	5.9	6.5	6.6	12.6* (9.2, 16.8)
Scanners per million population	3.12	3.10	··	3.50	··	··	4.26	4.70	4.50	5.9* (4.7, 7.2)
Poland										
Exams per scanner	··	··	··	1520.6	1747.8	1999.5	2435.3	1863.1	1742.8	2.3 (−9.0, 16.7)
Exams per 1000 population	··	··	··	1.1	1.2	1.4	1.5	1.6	1.5	7.0* (0.8, 14.3)
Scanners per million population	··	··	··	0.71	0.68	0.68	0.63	0.87	0.87	4.6 (−2.9, 12.7)
Slovakia										
Exams per scanner	806.0	1051.0	1077.3	1186.4	1115.6	1274.8	1304.4	1535.8	1672.5	7.6* (5.9, 9.9)
Exams per 1000 population	0.7	1.0	1.2	1.5	1.6	1.9	1.9	2.3	2.5	17.0* (15.6, 18.8)
Scanners per million population	0.93	0.92	1.11	1.29	1.47	1.47	1.47	1.47	1.47	7.4* (7.0, 7.8)
Spain										
Exams per scanner	1526.1	1448.8	1512.4	1613.0	1736.2	1885.2	2167.2	2331.8	2598.8	6.9* (6.4, 8.1)

Exams per 1000 population	2.1	2.1	2.3	2.6	2.8	3.2	3.7	4.2	4.6	10.7* (10.2, 11.7)
Scanners per million population	1.35	1.43	1.54	1.59	1.64	1.70	1.72	1.79	1.76	3.5* (3.3, 3.6)
Australia										
Exams per scanner	983.0	945.4	965.4	926.4	930.0	883.7	938.4	901.6	1037.7	0.3 (−0.2, 0.7)
Exams per 1000 population	1.4	1.7	2.0	2.2	2.5	2.7	3.1	3.5	3.9	13.4* (12.9, 14.1)
Scanners per million population	1.43	1.85	2.03	2.39	2.65	3.06	3.29	3.84	3.75	12.6* (11.2, 14.3)

Notes: Cells have two mid-dots (··) if no data are available; * indicates that the AAPC obtained from segmented regression analysis is significantly different from zero. In South Korea, the decrease in PET exams in 2015 is due to a change of payment standard for medical expenses. In Belgium, before 2016 PET activity was overestimated due to the partial inclusion of gamma camera activity. In France, before 2013 examinations practiced in private hospitals were wrongly compiled in ambulatory care, while during 2015 the official sources of data have been enhanced concerning all the equipment actually in use. In Poland, there was a change in data source in 2019 that led to the inclusion of specialty hospitals. In Australia, data relate to services rendered on a “fee-for-service” basis for which Medicare benefits were paid, which means that services that do not attract a Medicare benefit, such as those to public patients in hospitals or patients attending public Accident and Emergency Departments and public Outpatient Clinics, are excluded.
Abbreviations: OECD, Organisation for Economic Co-operation and Development; AAPC, average annual per cent change; CI, confidence interval
